# Analytical Solution for Chloride Diffusivity of Concrete with Aggregate Shape Effect

**DOI:** 10.3390/ma14143957

**Published:** 2021-07-15

**Authors:** Jian Zhang, Zhuo-Xuan Ying, Zhi-Wei Chen, Hai-Long Wang, Jian-Hua Li, Hui Yue, Jian-Jun Zheng

**Affiliations:** 1 Jiyang College, Zhejiang Agriculture and Forestry University, Zhuji 311800, China; JianZhang_zjyc@163.com (J.Z.); ZhuoxuanYing_jyc@163.com (Z.-X.Y.); 2 School of Civil Engineering and Architecture, Zhejiang University, Hangzhou 310012, China; 21812163@zju.edu.cn (Z.-W.C.); hailongwang_zju@163.com (H.-L.W.); 3 School of Civil Engineering and Architecture, Huzhou Vocational and Technical College, Huzhou 313000, China; xjbnljh@126.com; 4 School of Civil Engineering and Architecture, Zhejiang University of Technology, Hangzhou 310023, China; jjzheng_zjut@163.com

**Keywords:** concrete, chloride diffusivity, aggregate shape, analytical solution

## Abstract

In view of the key role of chloride diffusivity in evaluating concrete durability, it is very important to determine this parameter accurately by an effective approach. This paper establishes an analytical solution for chloride diffusivity of concrete that can consider the aggregate shape. In this approach, the aggregate shape is simulated as an ellipse and the equivalent model is applied to calculate the chloride diffusivity of equivalent aggregate composed of interface transition zone (ITZ) and aggregate. With resort to this model, at the meso scale, the concrete can be reduced from the original three-phase composition to the two-phase one (i.e., equivalent aggregates and cement paste). Based on the mesostructure of concrete that consisted of randomly dispersed equivalent elliptical aggregates and cement paste, the generalized Maxwell’s approach is formed to determine the chloride diffusivity of concrete. The corresponding chloride diffusion test is conducted and the thickness of ITZ is reasonably determined as 0.04 mm by SEM test. By comparing with the experimental data, the accuracy of the analytical solution is confirmed. Finally, the impact of aggregate shape on chloride diffusivity is discussed. The analytical results show that the chloride diffusivity has a reduction with the increase of aggregate content or decrease of aspect ratio.

## 1. Introduction

For civil engineering infrastructure located in a severe natural environment, the chloride diffusion coefficient is one of the important parameters to access the durability and design of reinforced concrete [1,2,3]. A great deal of engineering practice, laboratory research, and theoretical analysis have confirmed that the factors influencing the chloride ingression into concrete include the ratio of water to binder, the content and the shape of aggregate, and the interface transition zone (ITZ) [4,5,6]. Among them, the influence of aggregate shape on the chloride diffusion coefficient has been particularly focused on by scholars recently, especially through theoretical methods, because, testing and characterizing the influence of aggregate on the chloride diffusivity for concrete is a lengthy and tedious process [7]. Accordingly, alternative easy ways to obtain the chloride diffusivity have been the focus of research.

With the awareness of the importance of chloride diffusivity on the durability assessment of reinforced concrete structures, considerable research, both in experiment and in theory, have been carried out to investigate the chloride penetration in concrete. By means of through-diffusion technique and electro-migration method, the effects of aggregate content and ITZ on chloride diffusivity were investigated by Delagrave et al. [8]. Yang and Su used the electro-migration method to measure chloride diffusivity of concrete with various aggregate contents, and a simple analytical solution based on the single spherical aggregate model was proposed to quantitatively analyze the coupled influence of dilution effect and tortuosity effect of aggregate and the ITZ effect [9]. Caré et al. [10] studied the influence of aggregate content on the chloride diffusion characteristic in concrete by non-steady diffusion test. Based on the testing data, the analytical approach was formulated to establish the relationship between the ITZ and the chloride diffusivity of concrete. Zheng et al. [11] firstly used the electrical resistivity method to determine the chloride diffusivity of concrete and therefore, studied the influence of aspect ratio of aggregate and aggregate content on the chloride diffusivity of concrete based on the multi-layer concentric sphere model. Theoretical research mainly includes the analytical solution and the numerical method. Care and Herve [12] adopted a phase model to derive an analytical solution for effective diffusivity of concrete, which is the same as the multicoated sphere model proposed by Milton [13]. According to Garboczi and Bentz’s analytical method, concrete was firstly regarded as a composite sphere model composed of aggregate, ITZ divided by n layers and cement paste from the inner to the outer side. Then, the effective diffusion coefficient of concrete was calculated by transfer matrix method [14]. When the interfacial transition zone was assumed to be a homogeneous phase, by solving the Laplace diffusion equation, the three-phase composite model based on a circle [15] or sphere [16] was derived to evaluate the chloride diffusivity. Due to the non-uniform distribution of cement particles in the ITZ, the characteristics of ITZ show the gradient variation. By fitting the experimental results of cement particle distribution in the ITZ from Crumbie et al. [17], Zheng et al. [18] proposed a non-uniform ITZ model which was more consistent with the practical case. Based on the composite sphere model, an analytical approach was obtained to study the chloride diffusion coefficient. When considering the influence of aggregate shape, the differential effective medium theory was adopted to derive an analytical approximation for the chloride diffusivity of concrete by Zheng et al. [19]. Similarly, Choi et al. [20] recently developed a semi-empirical method to study the aggregate shape effect on the chloride penetration in concrete.

Since the aggregates in practical concrete mesostructure have different sizes according to the size distribution of aggregates, instead of a single aggregate model, the aggregate shape cannot satisfy the assumption of circle or sphere, as discussed in the above literatures. Actually, the shape of the natural aggregate (e.g., sand and gravel) model is closer to an ellipse in a two-dimensional case or an ellipsoid in a three-dimensional one. In recent years, numerical simulation methods have become the active research areas. The random walk simulation [21], finite element model [22], finite difference method [23], and lattice Boltzmann method [24] are widely used to study the chloride ingress into concrete. Based on the more efficient random walk algorithm (RWA) proposed by Kim and Torquato et al. [25,26], Zheng et al. [27] firstly reduced the three-phase concrete to the two-phase one by introducing equivalent aggregate, and; therefore, used the RWA to predict the chloride diffusivity. When considering the real shape of aggregate, Wang et al. [28] used Fourier series to simulate the profile of aggregate, and then the first passage theory suitable for pixel structure was utilized to simulate the chloride diffusion in concrete. Li et al. [29] developed two-phase finite element algorithm (FEA) models in both 2-D and 3-D to evaluate the effect of circular or spherical aggregate on the effective chloride diffusivity. Based on the concrete model with crushed granite and rounded gravel, the finite element model was applied by Jie et al. [30] to analyze the chloride transport in concrete. Liu et al. [31] established an artificial neural network to calculate the diffusion property of concrete. With resort to the finite element model, a numerical method for diffusivity of cracked concrete was proposed by Liu et al. [32]. Zhang et al. [24] established a multiscale simulation by a combination of the lattice Boltzmann model and the finite element method to predict the chloride diffusivity of concrete. The biggest advantage of this method was the improved simulation efficiency. To the best of our knowledge, the numerical simulation method is not only too time-consuming, but is also not easily utilized by engineers. Therefore, an analytical solution for predicting the chloride diffusion coefficient of concrete with consideration of the aggregate shape effect needs to be proposed.

In this paper, an experimental study is firstly conducted to discuss the impact of aggregate content on the chloride diffusion coefficient with a curing age up to 28 days. At the same time, an analytical solution is proposed to investigate the chloride diffusion regulation of concrete, and the accuracy is verified, in sequence, by the test data from literature and self-conducted experiment. Finally, the effect of aggregate shape on chloride diffusivity is evaluated.

## 2. Chloride Diffusion Test

The cement used for concrete specimen was Qianchao brand P.O 42.5 in Hangzhou city of China’s Zhejiang Province. Its chemical composition is listed in Table 1. The apparent density of the used cement was 3150 kg/m^3^. The pebble and natural sand were used. The Fuller gradation was adopted and therefore, the particle diameter of the aggregate was classified as seven intervals and ranged from 0.3 to 9.5 mm [33]. The proportion of fillers is listed in Table 2. The water/cement ratio (w/c) of concrete was selected as 0.5. To explore the percolation threshold caused by the ITZs connection, the designed aggregate content includes 0, 0.15, 0.2, 0.25, 0.3, 0.35, 0.4, 0.45, 0.5, 0.55, 0.6, 0.65, 0.7, and 0.75 in the previous investigation. It should be gently reminded that, in the literature [6], the aggregate contents of 0, 0.15, 0.25, 0.35, 0.45, 0.55, 0.65, and 0.75 are used. In this paper, the other data were adopted to verify the developed analytical solution. For the fixed water/binder ratio, the aggregate content C_a_ was varied from 0 to 0.7. The mix proportion is shown in Table 3. The water used for mixing was tap water from waterworks in Zhuji city. In order to avoid the calcium leaching of early age concrete specimens, the curing water was Ca(OH)_2_-saturated solution. The NaCl mass fraction of industrial salt used in laboratory for brine allocation before the conductivity test was greater than 99.1%.

**Figure 1:**
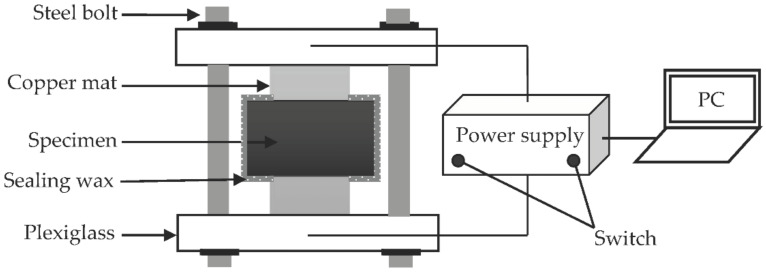
Schematic of test device.

The curing age *t* was set as 28 d. The initial size of the sample was a cylinder of Φ 100 mm × 100 mm. To minimize the accidental errors, three identical cylindrical specimens were made at a same mix ratio. After 28 d of standard curing, a double-blade cutter was adopted to sample the specimens with the size of Φ 100 mm × 50 mm, and the specimens were numbered and then immersed in Ca(OH)_2_-saturated solution at a curing temperature of 20 ± 2 °C. Finally, the chloride diffusion coefficient can be determined by an accelerated test [34]. The configuration of experimental equipment is displayed in Figure 1.

**Figure 2:**
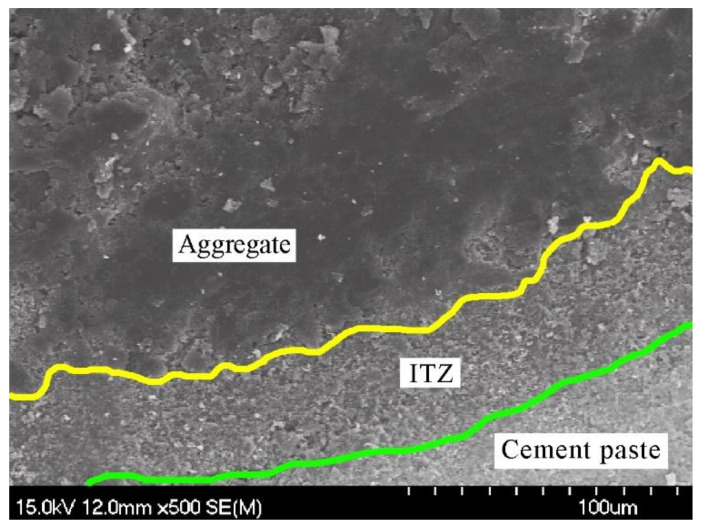
SEM image of ITZ [6]. (Reproduced with permission from Wang et al, Construction and Building Materials; published by Elsevier, 2021).

It should be noted that the thickness of the ITZ in previous theoretical studies was simply assumed. However, the thickness of ITZ had a great influence on the chloride diffusion characteristic in concrete. Therefore, it is necessary to determine the thickness of the interface transition zone by experimental investigation. In this study, the scanning electron microscopy (SEM) of Hitachi was used to detect the thickness of ITZ. The simple experimental steps mainly include: (1) A sample of 5 mm × 5 mm was obtained from the concrete specimen. (2) In order to avoid the influence of impurities on the surface of the sample on SEM detection, the sample should be washed by clean water. If the sample is filled with water, the water will vaporize violently in high vacuum, which not only affects the vacuum degree and pollutes the sample, but also destroys the microstructure of the sample. Thus, the sample should be baked in the oven at 50 °C for 10 h. (3) A thin gold layer was coated on the surface of sample. The testing result can be seen in Figure 2. The thickness of the ITZ can be easily determined as 40 μm, which was almost the same with the results of recent research [35,36].

The variation of D_c_ with C_a_ is listed in Table 4. It can be found from the experimental data that the chloride diffusivity measured by different specimens with the same mix proportion had deviation. The variances between the data from Specimen 1#, Specimen 2#, and Specimen 3# at different C_a_ were calculated as 0.05, 0.032, 0.006, 0.004, 0.006, 0.011, and 0.002. Thus, the reliability of the results of the studies was verified. Therefore, the validity of this chloride diffusion test was verified. In addition, D_c_ showed a regulation of analogous to linear reduction. The reason may be attributed that, the decrease effects induced by the dilute and tortuous of aggregate were obvious than the acceleration effect brought by ITZ [37]. After coupling of these three effects, the D_c_ happened to decrease linearly with the increase of C_a_. When the aggregate content increased from 0 to 0.7, the chloride diffusivity had a reduction of 45.7%.

## 3. Chloride Diffusivity of Concrete

To evaluate the influence of aggregate shape on chloride diffusivity, the shape of aggregate is simulated to be elliptical in the mesostructural model of concrete. The ITZ, a region of low strength and high porosity between the aggregate and the bulk cement paste, is formed by the low accumulation efficiency of cement particles near the aggregate. At present, a view with high recognition is the result obtained by Scrivener et al. [38] based on SEM observation, that is, the ITZ thickness is independent of the aggregate size. Therefore, the mesostructure of concrete can be assumed to be composed of a cluster of elliptical aggregates which formed by the specified gradation, an ITZ with a uniform thickness wrapped by the surface of aggregate, and bulk cement paste. D is denoted by the aggregate diameter (i.e., the size of minor axis or the sieving size). According to sieving analysis, the cumulative distribution function P(D) for Fuller distribution, in regards to the area of the aggregate, can be written as [39]

(1) $$P(D)=1-\frac{D_{\min}^{1.5}f(\alpha ,\beta )}{D^{1.5}f(\alpha_{0},\beta_{0})}$$

(2) $$f(\alpha ,\beta )=-0.4\cos^{1.5}\alpha \sin\alpha +0.4\sqrt{2}[2E(\beta ,1/\sqrt{2})-F(\beta ,1/\sqrt{2})]$$

(3) $$\alpha =\arccos(D/D_{\max}),{\;\alpha }_{0}=\arccos(D_{\min}/D_{\max})$$

(4) $$\beta =\arcsin[\sqrt{2}\sin(\alpha /2)],{\;\beta }_{0}=\arcsin[\sqrt{2}\sin(\alpha_{0}/2)]$$

where the D varies from D_min_ to D_max_; α, α_0_, β, and β_0_ are the parameters regarding to D, D_min_, and D_max_; E() and F() denote the Legendre’s integrals.

In order to simplify the three-phase composition of concrete to the two-phase one, the equivalent aggregate model must be introduced. Because the overlap degree of ITZs will increase with the increase of aggregate content, it should be pointed out that, according to the simulation results [40] when the aggregate content is more than 0.75, the overlapping area accounts for 10% of the total simulation area. Thus, the greater the overlap degree is, the smaller the equivalent ITZ thickness will be. Owing to the precondition that the improved Maxwell method used below does not permit overlapping between equivalent aggregates, reasonable determination of the equivalent ITZ thickness therein becomes critical. The detailed calculation process can be summarized into the following three steps:

Step 1. Determination of ITZ content f_i_ without ITZs overlapping. If the equivalent ITZ thickness is denoted as t_eq_, the ITZ content for an elliptical aggregate can be easily derived as

(5) $$A_{i}=2\mu E(\pi /2,\;\;\sqrt{1-1/\mu^{2}}{)\mathrm{Dt}}_{\mathrm{eq}}+{\pi t}_{\mathrm{eq}}^{2}$$

where μ represents the aspect ratio of elliptical aggregate. Thus, the ITZ volume fraction f_i_ is equal to

(6) $$\begin{array}{l} f_{i}=\int_{D_{\min}}^{D_{\max}}\frac{{4A}_{a}A_{i}}{\pi \mu \langle D^{2}\rangle }p(D)\mathrm{dD}\\ =\frac{4A_{a}}{\mu \pi \langle D^{2}\rangle }[2\mu E(\pi /2,\;\sqrt{1-1/\mu^{2}})\langle D\rangle t_{\mathrm{eq}}+2{\pi t}_{\mathrm{eq}}^{2}] \end{array}$$

where p() is probability density function of aggregate; 〈D〉 and $\langle D^{2}\rangle$ are the first and second moments of area, respectively. For any k moment, $\langle D^{k}\rangle$ can be expressed as

(7) $$\langle D^{k}\rangle =\int_{D_{1}}^{D_{N+1}}D^{k}p_{N}(D)\mathrm{dD}$$

Step 2. Monte Carlo method to determine the ITZ content f_i_ with ITZs overlap. First, the coordinates of random points in the simulation area with a square of a × a and the coordinates of x_i_ and y_i_ of point i should be determined, which can be expressed as

(8) $$x_{i}=\mathrm{ua}$$

(9) $$y_{i}=\mathrm{va}$$

where u and v is the random number between 0 and 1. Then, one needs to judge whether the random point i falls into the interior of the elliptical aggregates. If so, the number of random points N_a_ = N_a_ + 1; if not, the distance d_min_ from the random point i to the nearest surface of elliptical aggregate should be calculated as

(10) $$d_{\min}=\sqrt{\frac{{\left(\lambda_{\max}x_{i}\right)}^{2}}{{\left[\lambda_{\max}+{\left(\mu D/2\right)}^{2}\right]}^{2}}+\frac{{\left(\lambda_{\max}y_{i}\right)}^{2}}{{\left[\lambda_{\max}+{\left(D/2\right)}^{2}\right]}^{2}}}$$

(11) $$\frac{x^{2}}{{(\mu D)}^{2}}+\frac{y^{2}}{D^{2}}=\frac{1}{4}$$

(12) $$x=\frac{{(\mu D)}^{2}x_{i}}{4\lambda +{(\mu D)}^{2}}$$

(13) $$y=\frac{D^{2}y_{i}}{4\lambda +D^{2}}$$

where λ_max_ is the maximum real root by taking Equations (12) and (13) into Equation (11). If d_min_ is less than the ITZ thickness, the number of random points N_i_ = N_i_ + 1. Thus, the contents of aggregate and ITZ can be determined as

(14) $$f_{a}=\frac{N_{a}}{N}$$

(15) $$f_{i}=\frac{N_{i}}{N}$$

Step 3. Calculation of t_eq_. Based on Equations (6) and (15), h_eq_ can be obtained as

(16) $$h_{\mathrm{eq}}=\frac{\sqrt{b^{2}+4{\mathrm{af}}_{i}}-b}{2a}$$

where a and b are denoted as

(17) $$a=\frac{8A_{a}}{\mu \langle D^{2}\rangle }$$

(18) $$b=\frac{{8A}_{a}E(\pi /2,\sqrt{1-1/\mu^{2}})\langle D\rangle }{\pi \langle D^{2}\rangle }$$

It is found from Equations (16)–(18) that the influences of C_a_, p(D), and h on h_eq_ are significant.

**Figure 3:**
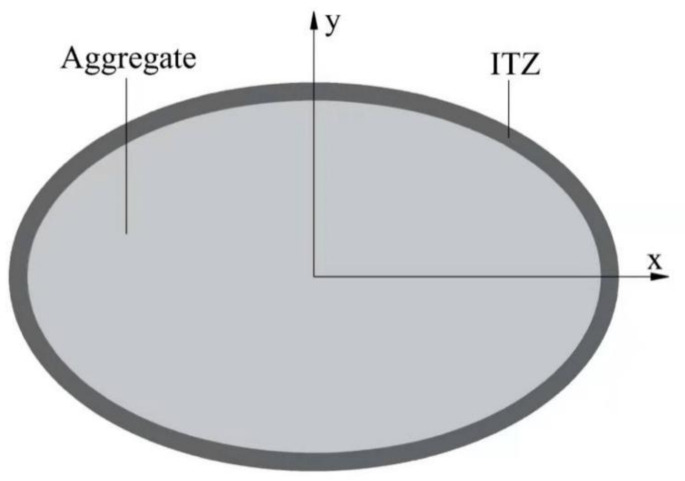
Equivalent elliptical aggregate coated with an equal thickness layer.

The ITZ is formed by the wall effect of cement particles on the aggregate surface, which has the characteristics of high porosity and low strength. In contrast, the porosity of aggregate is almost zero. Thus, in the mathematical modelling, the diffusivity of aggregate can be regarded as zero. According to Duan’s findings, the chloride diffusivities of the equivalent elliptical aggregate in the x- and y-directions, as shown in Figure 3, are given by [41].

(19) $$D_{x}=D_{i}+D_{i}\left[\frac{\left(1+\mu )A_{a}(D_{a}-D_{i}\right)}{\left(1+\mu )(A_{a}+A_{i})D_{i}+A_{i}(D_{a}-D_{i}\right)}\right]$$

(20) $$D_{y}=D_{i}+D_{i}\left[\frac{\left(1+\mu )A_{a}(D_{a}-D_{i}\right)}{\left(1+\mu )(A_{a}+A_{i})D_{i}+{\mu A}_{i}(D_{a}-D_{i}\right)}\right]$$

where D_a_ and D_i_ represent the diffusivities of aggregate and ITZ. Due to the compactness of aggregate, its diffusivity can be considered as zero. Thus, Equations (19) and (20) can be reduced as

(21) $$D_{x}=D_{i}+D_{i}\left[\frac{(1+\mu )A_{a}}{(1+\mu )A_{a}+{\mu A}_{i}}\right]$$

(22) $$D_{y}=D_{i}+D_{i}\left[\frac{(1+\mu )A_{a}}{(1+\mu )A_{a}+A_{i}}\right]$$

By averaging D_x_ and D_y_, D_ea_ can be expressed as

(23) $$D_{\mathrm{ea}}=\frac{1}{2}(D_{x}+D_{y})=D_{i}\left[1-\frac{{\left(1+\mu \right)}^{2}{(2A}_{a}+A_{i})A_{a}}{2[(1+\mu )A_{a}+{\mu A}_{i}][(1+\mu )A_{a}+A_{i}]}\right]$$

**Figure 4:**
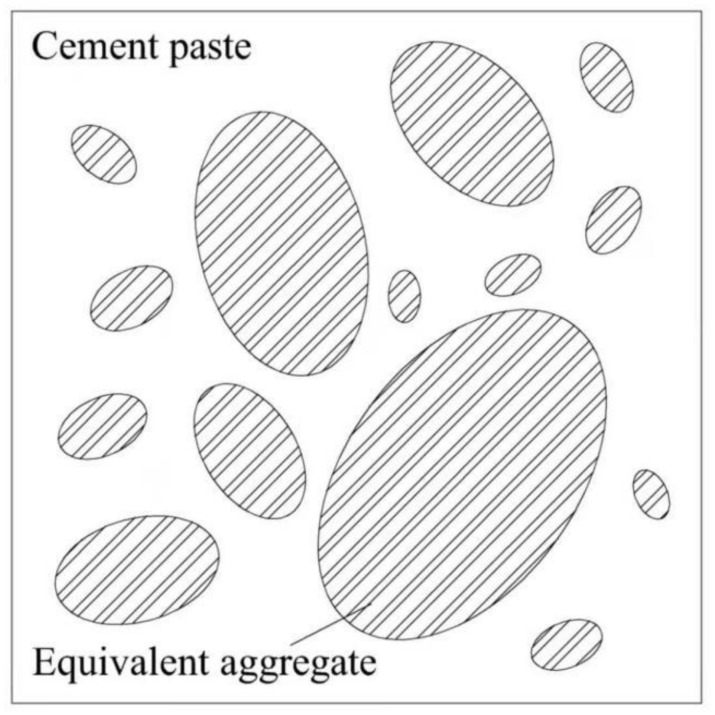
Two-phase concrete composed of bulk cement paste and equivalent aggregates.

With application of the equivalent aggregate, the diffusion problem of three-phase composite concrete can be simplified to a two-phase one (see in Figure 4). According to the Maxwell-Garnett approximation for two-dimensional case, the diffusivity of the two-phase composite concrete with circular aggregate as inclusions can be given by:

(24) $$\frac{D_{c}-D_{a}}{D_{c}+D_{a}}=(1-f_{1})\frac{D_{\mathrm{cp}}-D_{a}}{D_{\mathrm{cp}}+D_{a}}$$

where D_cp_ is the chloride diffusivity of cement paste, and f_1_ is the content of equivalent aggregate.

The extendibility of the Maxwell method for a macroscopically anisotropic concrete composed of M-1 equivalent elliptical aggregate with the same aspect ratio can be expressed as [42]:

(25) $$\sum_{j=1}^{M}f_{j}(D_{c}-D_{\mathrm{ea},j})R^{j1}=0$$

where *D*_c_ is the effective diffusion tensor, *D*_ea,j_ = *I* × D_ea,j_.

(26) $$R^{j1}={\left[I+A^{*}\frac{D_{\mathrm{ea},j}-D_{\mathrm{ea},1}}{D_{\mathrm{ea},1}}\right]}^{-1}$$

(27) $$A^{*}=\left[\begin{array}{cc} Q & 0\\ 0 & 1-Q \end{array}\right]$$

(28) $$Q=\frac{1}{2}\left\{1+\frac{1}{\mu^{2}-1}\left[1-\frac{1}{{2\chi }_{b}}\ln⟮\frac{1+\chi_{b}}{1-\chi_{b}}⟯\right]\right\}$$

(29) $$\chi_{b}=\sqrt{1-\frac{1}{\mu^{2}}}$$

(30) $$f_{j}=\frac{C_{a}D_{\mathrm{ea},j}^{2}\mu +A_{i,j}}{\sum_{j=1}^{M-1}{(D}_{\mathrm{ea},j}^{2}\mu +A_{i,j})}$$

here, *I* is the second-order identity matrix; A* represents the depolarization tensor; A_i,j_ is the ITZ area of the j-th aggregate.

It should be noted that, if the elliptical aggregates are randomly distributed, then *R*^jl^ in the Equation (26) should be rewritten as

(31) $$R^{j1}=\frac{R^{j1}}{2}I$$

Finally, by solving Equations (25)–(31), the chloride diffusivity of concrete D_c_ can be derived.

## 4. Experimental Verification and Discussions

**Figure 5:**
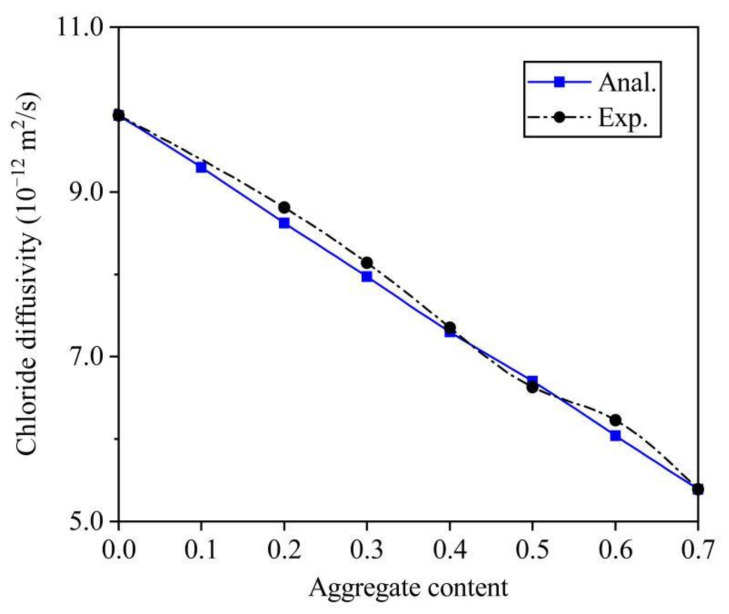
Comparison with self-conducted test data.

In order to verify the accuracy of this analytical solution, two groups of data from different experiments were chosen. The first group of experimental data was collected from self-conducted experiment. Before the analytical results made a comparison of experimental data, some parameters (e.g., the thickness t and the chloride diffusivity D_i_ of ITZ, the aspect ratio of aggregate μ) needed to be determined in advance. According to the result of our self-conducted SEM, the t was reasonably determined as 0.04 mm. Through analyzing ten different shapes of aggregates, the average value of μ was resolved as 2.02 [43]. As for D_i_, there is a lack of a direct test method to determine the chloride diffusivity of ITZ at present. Herein, the back-calculating manner was used. Based on the D_cp_ = 9.93 × 10^−12^ m^2^/s at C_a_ = 0 and D_c_ = 5.39 × 10^−12^ m^2^/s at C_a_ = 0.7, D_i_ was calibrated as 4.06 × 10^−11^ m^2^/s. After gathering these parameters, the D_c_ at an arbitrary C_a_ can be computed by this analytical solution (see in Figure 5). The relative errors between the computed results and test data were 2.2, 2.1, 2.1, 0.68, and 1.1%, at C_a_ varied from 0.2 to 0.6. There is not obvious permeation effect (i.e., once the aggregate content exceeds a certain value, the chloride diffusivity will increase significantly) is found either in the simulation results or in the experimental values. The reason may be due to the difference of the mechanism of diffusion and permeation. The effect of permeation threshold on diffusion is much less than that on permeability. In addition, the chloride diffusion coefficient decreases linearly with the increase of aggregate content, which is mainly due to the acceleration effect of ITZs, dilution and zigzag effects caused by aggregates.

**Figure 6:**
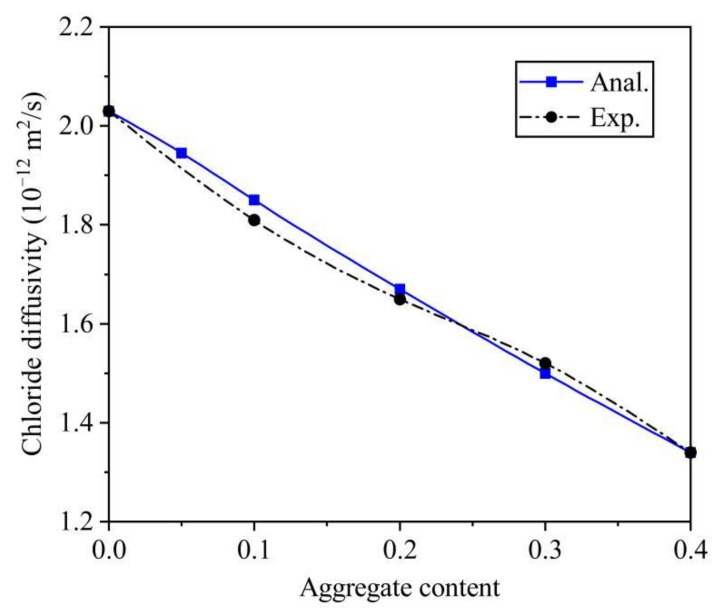
Comparison with test data from Yang and Su.

In addition, the test data from Yang and Su [9] was collected. In the experiment, the ASTM I cement was used and the w/c was 0.4. The C_a_ was varied from 0 to 0.4, and the dosages of fly ash and slag were 0.1 and 0.2 times the weight of cement. The D_max_ and D_min_ were 9.5 and 0.15 mm. At the aggregate grading intervals of [0.15, 0.3], [0.3, 0.6], [0.6, 1.18], [1.18, 2.36], [2.36, 4.75], and [4.75, 9.5], the aggregate content was approximate 0.03, 0.08, 0.29, 0.28, 0.24, and 0.08, respectively. The curing conditions of the specimens were standard curing. After annual curing, the D_c_ was determined by an accelerated test scheme. With the same as the first validation, the identical t and μ were used. The D_i_ is calibrated as 9.5 × 10^−12^ m^2^/s by D_c_ = 1.34 × 10^−12^ m^2^/s at C_a_ = 0.4. The simulation results and the test data can be seen in Figure 6. The relative errors between them were 2.2, 1.2, and 1.3% at C_a_ varied from 0.1 to 0.6. Therefore, the accuracy of the analytical solution was fully verified.

**Figure 7:**
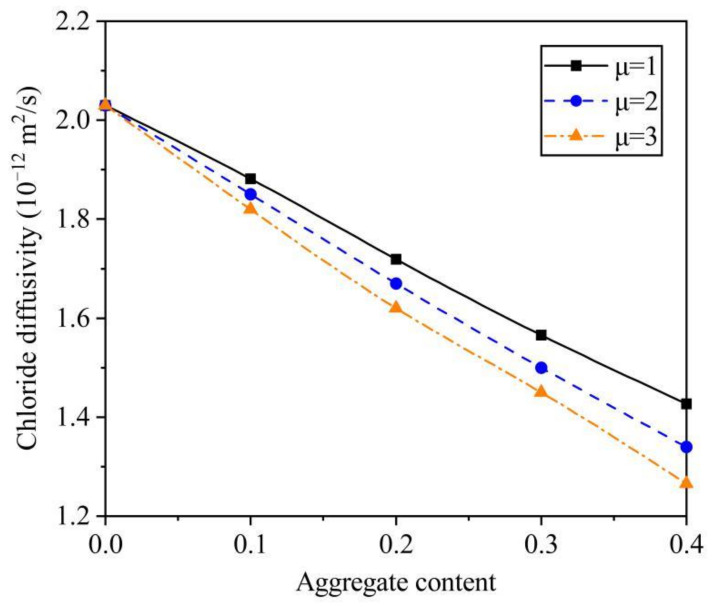
The impact of μ on D_c_.

With consideration of the impact of aggregate shape (e.g., μ) on D_c_, a sensitivity analysis was performed. It should be pointed out that, all the parameters, but aspect ratio, were the same as the second set of validation. For D_i_ = 9.5 × 10^−12^ m^2^/s, h = 0.04 mm, μ = 1, 2, and 3, the variations of D_c_ with C_a_ and μ can be seen in Figure 7. For C_a_ = 0.1, 0.2, 0.3, and 0.4, when μ decreased from 3 to 1, D_c_ was increased by 3.4%, 6.1%, 8.0%, and 12.8%, respectively. For μ = 1, 2, and 3, when C_a_ was up from 0.1 to 0.4, D_c_ has a reduction of 31.9%, 38.1%, and 43.9%, respectively.

## 5. Conclusions

The analytical solution for predicting chloride diffusivity of concrete was established in this paper. To verify the accuracy of the developed method, the corresponding experiments were performed. Finally, the main influence of the aspect ratio on the chloride diffusivity of concrete was quantified. The main conclusions of this study are as follows:

1. By comparison of the data from the self-conducted experiment and the literature, the average relative error is only 1.64% and 1.57%, respectively. Thus, the accuracy of the analytical solution was fully verified.
2. With resort to the SEM observation, the thickness of ITZ was reasonably determined as 0.04 mm. In addition, based on the conductivity method, the chloride diffusivities with various aggregate contents were measured. It can be found that D_c_ approximately changed linearly with C_a_, which was mainly due to the coupled effects from the acceleration effect of ITZs and the dilution and zigzag effects caused by aggregates. When the aggregate content increased from 0 to 0.7, the chloride diffusivity had a reduction of 45.7%.
3. According to the sensitivity analysis based on the analytical solution, the impact of aggregate shape on D_c_ was significant. For C_a_ = 0.1, 0.2, 0.3, and 0.4, when μ decreased from 3 to 1, D_c_ was increased by 3.4%, 6.1%, 8.0%, and 12.8%, respectively. The main reason can be analyzed as the increase of μ leading to the decrease of ITZ content and the increase of the tortuosity of aggregate.

## Figures and Tables

**Table 1:** Chemical compositions of cement.

Material	CaO (%)	SiO_2_ (%)	Al_2_O_3_ (%)	Fe_2_O_3_ (%)	MgO (%)	SO_3_ (%)	K_2_O (%)	Na_2_O (%)
Cement	65.31	21.21	3.88	3.25	1.12	0.98	0.57	0.12

**Table 2:** The proportion of fillers.

**Aggregate Size (mm)**	0.3–0.6	0.6–1.18	1.18–2.36	2.36–4.75	4.75–9.5
**Proportion**	9.0%	12.2%	17.8%	25.4%	35.6%

**Table 3:** Mix proportion of concrete at w/c = 0.5.

C_a_	Dosage of Material (kg/m^3^)		
	Cement	Water	Aggregate
0	1223.3	611.7	0
0.2	978.6	489.3	536
0.3	856.3	428.2	804
0.4	734.0	367	1072
0.5	611.7	305.8	1340
0.6	489.3	244.7	1608
0.7	367.0	183.5	1876

**Table 4:** The D_c_ measured by experiment.

*C_a_*	Specimen 1# (10^−12^ m^2^/s)	Specimen 2# (10^−12^ m^2^/s)	Specimen 3# (10^−12^ m^2^/s)	Average Value (10^−12^ m^2^/s)
0	10.13	9.97	9.69	9.93
0.2	8.72	8.96	9.07	8.92
0.3	8.17	8.05	8.2	8.14
0.4	7.28	7.36	7.41	7.35
0.5	6.62	6.71	6.56	6.63
0.6	6.23	6.12	6.33	6.23
0.7	5.35	5.38	5.43	5.39

## Data Availability

Data sharing not applicable.

## References

[1] Petcherdchoo A. (2016). Pseudo-coating model for predicting chloride diffusion into surface-coated concrete in tidal zone: Time- dependent approach. Cem. Concr. Compos..

[2] Zhang J., Zhou X.Z., Zheng J.J., Ye H.L., Yang J. (2020). Experimental investigation and analytical modeling of chloride diffusivity of fly ash concrete. Materials.

[3] Liu Q.F., Hu Z., Lu X.Y., Yang J., Azim I., Sun W.Z. (2020). Prediction of chloride distribution for offshore concrete based on statistical analysis. Materials.

[4] Liu Q.F., Easterbrook D., Yang J., Li L.Y. (2015). A three-phase, multi-component ionic transport model for simulation of chloride penetration in concrete. Eng. Struct..

[5] Liu Q.F., Feng G.L., Xia J., Yang J., Li L.Y. (2018). Ionic transport features in concrete composites containing various shaped aggregates: A numerical study. Compos. Struct..

[6] Wang H.L., Chen Z.W., Sun X.Y., Zhang J., Zheng J.J. (2021). New numerical method for predicting chloride diffusivity of concrete considering the profiles of practical aggregates. Constr. Build. Mater..

[7] Liu Y., Presuel-Moreno F.J., Paredes M.A. (2015). Determination of chloride diffusion coefficients in concrete by electrical resistivity method. ACI Mater. J..

[8] Delagrave A., Bigas J.P., Ollivier J.P., Marchand J., Pigeon M. (1997). Influence of the interfacial zone on the chloride diffusivity of mortars. Adv. Cem. Based Mater..

[9] Yang C.C., Su J.K. (2002). Approximate migration coefficient of interfacial transition zone and the effect of the aggregate content on the migration coefficient of mortar. Cem. Concr. Res..

[10] Caré S. (2003). Influence of aggregates on chloride diffusion coefficient into mortar. Cem. Concr. Res..

[11] Zheng J.J., Zhou X.Z., Huang X.F., Fu C.Q. (2014). Experiment and modeling of the effect of aggregate shape on the chloride diffusivity of concrete. J. Mater. Civ. Eng..

[12] Caré S., Hervé H. (2004). Application of a n-phase model to the diffusion coefficient of chloride in mortar. Transp. Porous Media.

[13] Milton G.W. (1981). Concerning bounds on the transport and mechanical properties of multicomponent composite materials. Appl. Phys. A Mater..

[14] Garboczi E.J., Bentz D.P. (1997). Analytical formulas for interfacial transition zone properties. Adv. Cem. Based Mater..

[15] Zheng J.J., Zhou X.Z. (2007). Prediction of the chloride diffusion coefficient of concrete. Mater. Struct..

[16] Zheng J.J., Zhou X.Z. (2008). Three-phase composite sphere model for the prediction of chloride diffusivity of concrete. J. Mater. Civ. Eng..

[17] Crumbie A.K. (1994). Characterisation of the Microstructure of Concrete. Ph.D. Thesis.

[18] Zheng J.J., Hong S.W., Buenfeld N.R. (2009). Assessing the influence of ITZ on the steady-state chloride diffusivity of concrete using a numerical model. Cem. Concr. Res..

[19] Zheng J.J., Zhou X.Z., Xing H.Y., Jin X.Y. (2015). Differential effective medium theory for the chloride diffusivity of concrete. ACI Mater. J..

[20] Choi Y.C., Park B., Pang G.S., Lee K.M., Choi S.C. (2017). Modelling of chloride diffusivity in concrete considering effect of aggregates. Constr. Build. Mater..

[21] Ma H.Y., Hou D.S., Li Z.J. (2015). Two-scale modeling of transport properties of cement paste: Formation factor, electrical conductivity and chloride diffusivity. Comp. Mater. Sci..

[22] Du X.L., Jin L., Ma G.W. (2014). A meso-scale numerical method for the simulation of chloride diffusivity in concrete. Finite Elem. Anal. Des..

[23] Ukrainczyk N., Koenders E. (2014). Representative elementary volumes for 3D modeling of mass transport in cementitious materials. Model. Simul. Mater. Sci. Eng..

[24] Zhang M.Z., Ye G., Breugel K.V. (2014). Multiscale lattice Boltzmann-finite element modelling of chloridediffusivity in cementitious materials. Part I: Algorithms and implementation. Mech. Res. Commun..

[25] Kim I.C., Torquato S. (1990). Determination of the effective conductivity of heterogeneous media by Brownian motion simulation. J. Appl. Phys..

[26] Kim I.C., Torquato S. (1991). Effective conductivity of suspensions of hard spheres by Brownian motion simulation. J. Appl. Phys..

[27] Zheng J.J., Zhang C.Y., Sun L.Z., Zhou X.Z. (2016). A Brownian motion simulation for the chloride diffusivity of concrete. Constr. Build. Mater..

[28] Wang L.B., Wang X.R., Mohammad L., Abadie C. (2005). Unified method to quantify aggregate shape angularity and texture using Fourier analysis. J. Mater. Civ. Eng..

[29] Li L.Y., Xia J., Lin S.S. (2012). A multi-phase model for predicting the effective diffusion coefficient of chlorides in concrete. Constr. Build. Mater..

[30] Jie W., Dassekpo J.B.M., Wan C.Y., Zha X.X. (2017). Experimental and numerical modeling of chloride diffusivity in hardened cement concrete considering the aggregate shapes and exposure-duration effects. Results Phys..

[31] Liu Q.F., Iqbal M.F., Yang J., Lu X.Y., Zhang P., Rauf M. (2021). Prediction of chloride diffusivity in concrete using artificial neural network: Modelling and performance evaluation. Constr. Build. Mater..

[32] Liu Q.F., Yang J., Xia J., Easterbrook D., Li L.Y., Lu X.Y. (2015). A numerical study on chloride migration in cracked concrete using multi-component ionic transport models. Comp. Mater. Sci..

[33] Zheng J.J. (2000). Mesostructure of Concrete-Stereological Analysis and Some Mechanical Implications.

[34] Lu X.Y. (1997). Application of the Nernst-Einstein equation to concrete. Cem. Concr. Res..

[35] Fu C.Q., Ling Y.F., Wang K.J. (2020). An innovation study on chloride and oxygen diffusions in simulated interfacial transition zone of cementitious material. Cem. Concr. Compos..

[36] Zhang H.R., Ji T., Liu H. (2019). Performance evolution of the interfacial transition zone (ITZ) in recycled aggregate concrete under external sulfate attacks and dry-wet cycling. Constr. Build. Mater..

[37] Sun G.W., Zhang Y.S., Sun W., Liu Z.Y., Wang C.H. (2011). Multi-scale prediction of the effective chloride diffusion coefficient of concrete. Constr. Build. Mater..

[38] Scrivener K.L., Nemati K.M. (1996). The percolation of pore space in the cement paste/aggregate interfacial zone of concrete. Cem. Concr. Res..

[39] Zheng J.J., Li C.Q., Zhao L.Y. (2003). Simulation of two-dimensional aggregate distribution with wall effect. J. Mater. Civ. Eng..

[40] Zheng J.J., Zhang J., Zhou X.Z., Song W.B. (2018). A numerical algorithm for evaluating the chloride diffusion coefficient of concrete with crushed aggregates. Constr. Build. Mater..

[41] Duan H.L., Karihaloo B.L., Wang J.Q., Yi X. (2006). Effective conductivities of heterogeneous media containing multiple inclusions with various spatial distributions. Phys. Rev. B.

[42] Torquato S. (2001). Random Heterogenerous Materials: Microstructure and Macroscopic Properties.

[43] Zheng J.J., Zhang J., Zhou X.Z., Wang J.W., Ding Y.W. (2020). Random walk algorithm for chloride diffusivity of concrete. ACI Mater. J..

